# Comparison Between Linear Stapler and Circular Stapler After Laparoscopic-Assisted Distal Gastrectomy in Patients With Gastric Cancer

**DOI:** 10.3389/fsurg.2022.858236

**Published:** 2022-05-06

**Authors:** Danping Sun, Renhua Zhang, Meng Wei, Peng Liu, Xin Zhong, Yize Liang, Yuanyuan Chen, Yadi Huang, Wenbin Yu

**Affiliations:** ^1^Department of Gastrointestinal Surgery, General Surgery, Qilu Hospital, Cheeloo College of Medicine, Shandong University, Jinan, China; ^2^Outpatient Department, Qilu Hospital, Cheeloo College of Medicine, Shandong University, Jinan, China; ^3^Department of Nursing Department, Qilu Hospital, Cheeloo College of Medicine, Shandong University, Jinan, China

**Keywords:** gastric cancer, laparoscopic distal gastrectomy, Billroth II + Braun anastomosis, linear stapler, circular stapler

## Abstract

**Background and Aim:**

To evaluate the safety and efficacy of laparoscopy distal gastrectomy using a linear stapler compared with a circular stapler in patients with gastric cancer.

**Methods:**

We retrospectively reviewed 173 patients who underwent laparoscopic distal gastrectomy for gastric cancer at a single center from January 2018 to December 2020. Patients were categorized into the linear stapler group and the circular stapler group. General data, intraoperative and postoperative outcomes, postoperative pathological results, postoperative complications, and postoperative follow-up in the two groups were compared and analyzed.

**Results:**

The operation time (208.76 ± 32.92 vs. 226.69 ± 26.92 min, *p* < 0.05), anastomosis time (71.87 ± 9.50 vs. 90.56 ± 3.18 min, *p* < 0.05), time to first flatus (68.60 ± 25.96 vs. 76.16 ± 21.05 h, *p* < 0.05), time to the first sip of water (3.66 ± 0.61 vs. 4.07 ± 0.77 days, *p* < 0.05), and time to the first liquid diet (4.43 ± 1.02 vs. 5.03 ± 1.70 days, *p* < 0.05) were significantly shorter in the linear stapler group. In addition, the highest postoperative body temperature within 3 days (37.4 ± 0.61 vs. 37.7 ± 0.61, *p* < 0.05) after the operation, white blood cell count (WBC) on the 3rd day (9.07 ± 2.52 vs. 10.01 ± 2.98 × 10∧9/L, *p* < 0.05), and average gastric tube drainage within 3 days (36.65 ± 24.57 vs. 52.61 ± 37 ml, *p* < 0.05) were also significantly lower in the linear stapler group.

**Conclusions:**

Both circular and linear staplers are safe and feasible for gastrointestinal reconstruction in laparoscopic distal gastrectomy. In contrast, a linear stapler has advantages over a circular stapler in shortening operation time and accelerating the postoperative recovery of patients.

## Introduction

Gastric cancer is the fifth most frequently diagnosed cancer and the fourth leading cause of cancer mortality worldwide, and the highest incidence rates are reported in Eastern and Western Asia (1). In addition, gastric cancer is the second most common malignancy and the second leading cause of cancer-related death in China (2). With the development of basic research and clinical trials, the clinical treatment of gastric cancer has developed from a simple surgical treatment to a comprehensive therapy based on surgery, such as chemotherapy, radiotherapy, immunotherapy, and targeted therapy (3). In 1994, Kitano et al. (4) first reported laparoscopy-assisted distal gastrectomy (LADG) for gastric cancer, which opened a new era by applying laparoscopic technology in gastrointestinal surgery. Patients and clinicians have widely favored laparoscopic surgery due to its advantages, such as a clear surgical field, less trauma, light postoperative pain, and fast postoperative recovery (5–7).

Treatments for gastric cancer include abdominal surgery, laparoscopic surgery, and endoscopic submucosal dissection (8), which are primarily dependent on the patient's health and the stage of the tumor. In laparoscopic radical gastric cancer surgery, the choice of surgical method depends on the location of the primary tumor, the depth of tumor invasion, and the ease of operation (9–11). According to the Japanese “Regulations for the Treatment of Gastric Cancer,” radical distal gastrectomy with D2 lymph node dissection has been recommended as the standard surgical procedure for patients with a lower one-third of the stomach (12, 13). The methods of digestive tract reconstruction after distal gastrectomy with D2 lymph node dissection included Billroth I, Billroth II + Braun, and Roux-en-Y (14). Among all anastomosis methods, Billroth II + Braun anastomosis is the most favored by gastric surgeons (15). With the rapid development of both laparoscopic surgery technology and laparoscopic instruments, anastomotic instruments have been widely used in laparoscopic gastric cancer surgery (16). Presently, the two commonly used anastomosis instruments are circular and linear staplers (17). Although sufficient research has shown the feasibility and tumor safety of laparoscopic distal gastrectomy, no consensus has been reached on the preferred stapler of reconstruction (18).

Therefore, we compared and analyzed the general data, intraoperative and postoperative outcomes, postoperative pathological results, postoperative complications, and postoperative follow-up of patients with linear and circular anastomosis and evaluated the application value of linear staplers and circular staplers in laparoscopic radical distal gastrectomy (Billroth II + Braun anastomosis) to finally determine the preferred anastomosis method.

## Materials and Methods

### Study Design and Patients

The clinical and postoperative data of 173 patients with gastric cancer who underwent laparoscopic-assisted radical distal gastrectomy at Qilu Hospital of Shandong University from January 2018 to December 2020. Among them, 93 patients underwent Billroth II + Braun anastomosis with the linear stapler, and 80 patients underwent Billroth II + Braun anastomosis with the circular stapler.

Inclusion criteria: (1) patients diagnosed with gastric cancer preoperatively by gastroscopy and pathology; (2) a preoperative intensive CT assessment of no distant metastasis and no involvement of the duodenum; (3) intraoperative laparoscopic-assisted radical distal gastrectomy; (4) there were no apparent contraindications in the relevant auxiliary examinations before the operation.

Exclusion criteria: (1) patients who received neoadjuvant chemotherapy before operation (*n* = 67); (2) patients with distant metastases in the abdominal cavity and unable to achieve radical surgery; (3) patients with severe comorbidities (such as heart, lung, kidney, and other diseases) who are unable to tolerate laparoscopic operation; (4) emergency surgery due to the complication (bleeding, obstruction, or perforation) caused by gastric cancer; (5) patients with a history of upper abdominal surgery; and (6) patients with other malignant tumors.

### Surgical Technique

#### Laparoscopic Distal Gastrectomy With Lymph Node Dissection

All operations were performed by the Gastrointestinal Surgery Team of Qilu Hospital of Shandong University. All patients completed laparoscopic-assisted radical distal gastrectomy by the 15th edition of the Japanese gastric cancer management protocol. After general anesthesia, the patients were placed in a horizontal supine position, and their legs were separated. The chief surgeon stood on the patient's left side, the first assistant stood on the right, and the second assistant stood between the patient's legs to manipulate the laparoscope. The standard five-hole method was performed. After D2 lymph node dissection and complete dissection, the duodenum was cut off from 3 cm below the pyloric sphincter, and the stomach was cut off at the proximal 5 cm of the tumor. The gastric stump was stitched intermittently with a 3-0 silk thread to stop bleeding, and the duodenal stump with 4-0 prolene sutures was continuously sutured to strengthen the duodenum stump. The anastomosis was completed after a 6 cm incision was made in the middle of the upper abdomen. In both groups, a 60 cm gastric tube was placed after the operation for postoperative drainage and the monitoring of gastric acidity (pH), a naso-intestinal nutrition tube was put into the small intestine of the output loop, and enteral nutrition was started on the 3rd postoperative day.

#### Linear Anastomosis

**Figure 1:**
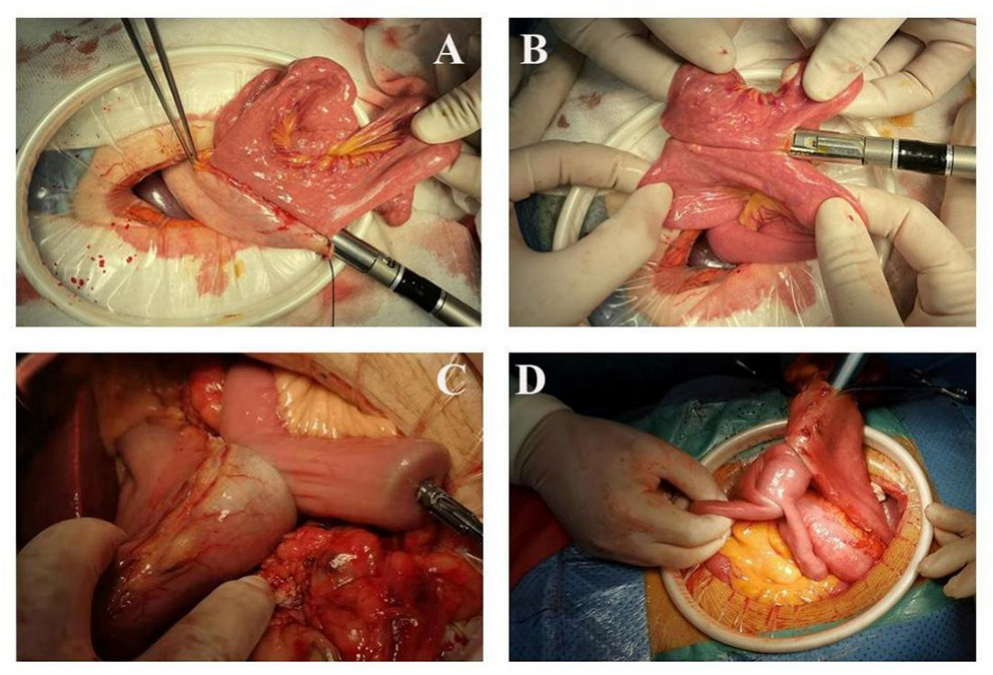
Laparoscopic-assisted distal gastrectomy **(A,B)** Linear stapler anastomosis; **(C,D)** Circular stapler anastomosis.

As shown in Figures 1A,B, the intestine before the colon was lifted 20 cm distal to the Treitz ligament. Small incisions (0.5 cm) were made at the greater curvature of the remnant stomach and the distal jejunum, and then both arms of the 60 mm linear Endo-GIA stapler were inserted into the small incision to close the distal jejunum and the remnant stomach and complete the side-to-side anastomosis of the gastrojejunal. The width of the anastomosis was 50~60 mm, with absorbable sutures and continuous sutures to close the common opening. A similar method was used to complete the side-to-side anastomosis of the jejunum and jejunum (Braun anastomosis) at 10 cm from the gastrointestinal anastomosis. The width of the Braun anastomosis was 4 cm, and the anastomosis, digestive tract stump, etc., were reinforced with intermittent sutures with the 3-0 V-Loc suture.

#### Circular Anastomosis

As shown in Figures 1C,D, the jejunum was lifted 20 cm from the ligament of Treitz and raised in the front of the colon. A purse-string suture (not tightened) was performed on the contralateral mesangial border of the jejunum, the intestinal wall of the suture was cut, and a nail anvil was inserted to pull the purse-string to complete nail anvil placement. After opening the gastric wall on the greater curvature of the stomach, a 25 mm stapler was inserted. The posterior gastric wall was anastomosed with the raised jejunum ~5 cm proximal to the tumor. The nail anvil was placed in one intestinal tube in the same way at a distance of 10 cm from the gastrointestinal anastomosis. A 25 mm stapler was inserted through the gastrointestinal anastomosis to the corresponding position of the other side of the intestine to complete the side-to-side anastomosis of the jejunum and jejunum (Braun anastomosis). Finally, the distal gastric wall was cut off very close to the gastrojejunal anastomosis, and the gastric end was closed. The anastomosis, digestive tract stump, etc., were reinforced with intermittent sutures with the 3-0 V-Loc suture.

### Observation Index and Evaluation Standard

#### Observation Indicators

(1) Intraoperative observation and recording indicators: operation time, tissue dissection time, anastomosis time, and intraoperative blood loss.
(2) Short-term postoperative observation indicators: time to first flatus, time to the first sip of water, time to a first liquid diet, procalcitonin (PCT) on the 3rd day, WBC on the 3rd day, the highest body temperature within 3 days, the pH value of gastric acidity, gastric drainage, and hospital stay.
(3) Postoperative complications: anastomosis-related complications (anastomotic bleeding, anastomotic leakage, and anastomotic stenosis), functional complications (delayed gastric emptying, dumping syndrome, and inflammatory intestinal obstruction), and others (pneumonia, cholecystitis, and lymphatic leakage).
(4) Postoperative pathological results: tumor length, number of lymph nodes harvested, number of positive lymph nodes, distance from proximal resection margin to the tumor, distance from distal resection margin to the tumor, T stage, M stage, and TNM stage of the tumor.
(5) Postoperative follow-up: anastomotic width, average number of meals, weight change, symptoms of reflux, proton pump inhibitor (PPI) medication, and dumping syndrome.

#### Evaluation Index

(1) Operation-related complications were recorded according to the Clavien–Dindo grading standards (I: Acceptable treatments are antiemetics, antipyretics, analgesics, diuretics, electrolytes, and physical therapy; II: Blood transfusions, total parenteral nutrition, and medications other than such allowed for grade I complications; III: Complications required surgical, endoscopic, or radiological intervention; IV: Life-threatening complications requiring ICU admission; V: Death) (19); (2) pathological staging was performed according to 2020 NCCN gastric cancer guidelines for TNM staging; and (3) endoscopic findings were analyzed using the residue, gastritis and bile (RGB) classification, and reflux esophagitis was evaluated using the Los Angeles classification.

### Statistical Analysis

All variables were analyzed by SPSS 26.0 software, continuous variables were expressed as average ± SD, metrological variables were tested by *t*-test, and classified variables were tested by χ^2^ test and Fisher's accurate test. The difference was statistically significant (*p* < 0.05).

## Result

### General Data

As shown in Table 1, there were no significant differences in age (58.92 ± 10.36 vs. 60.36 ± 10.38 years), sex (*p* = 0.784), body mass index (BMI) (24.56 ± 3.16 vs. 23.96 ± 3.32, *p* = 0.228), preoperative ASA score (*p* = 0.304) or comorbidities (*p* = 0.946) between the linear stapler anastomosis group and the circular stapler anastomosis group.

**Table 1:** General data of patients who underwent laparoscopic distal gastrectomy using linear stapler and circular stapler.

**Index**	**Linear anastomosis** **(*n* = 93)**	**Circular anastomosis** **(*n* = 80)**	***p*** **-value**
Age (years)	58.92 ± 10.36	60.36 ± 10.38	0.365
Sex			0.784
Male	68	57	
Female	25	23	
BMI (kg/m2)	24.56 ± 3.16	23.96 ± 3.32	0.228
**ASA score**
1	17	12	0.304
2	67	54	
3	9	14	
**Comorbidity**
Hypertension	23	12	
Diabetes mellitus	11	4	0.946
Coronary heart disease	7	4	
Others	11	5	

### Intraoperative and Postoperative Outcomes

As shown in Table 2, the differences in blood loss (28.66 ± 6.70 vs. 30.76 ± 8.18 ml) and tissue dissection time (84.19 ± 11.46 vs. 86.53 ± 7.93 min) between the two groups were not statistically significant (*p* > 0.05). The operation time (208.76 ± 32.92 vs. 226.69 ± 26.92 min, *p* < 0.001) and anastomosis time (71.87 ± 9.50 vs. 90.56 ± 3.18 min, *p* < 0.001) were significantly shorter in the linear stapler group than in the circular stapler group.

**Table 2:** Intraoperative and postoperative outcomes of patients who underwent laparoscopic distal gastrectomy using linear stapler and circular stapler.

**Index**	**Linear anastomosis (*n* = 93)**	**Circular anastomosis (*n* = 80)**	***p*** **-value**
Operation time (min)	208.76 ± 32.92	226.69 ± 26.92	<0.001
Tissue dissection time (min)	84.19 ± 11.46	86.53 ± 7.93	0.128
Anastomosis time (min)	71.87 ± 9.50	90.56 ± 3.18	<0.001
Intraoperative blood loss (ml)	28.66 ± 6.70	30.76 ± 8.18	0.064
Time to first flatus (h)	68.60 ± 25.96	76.16 ± 21.05	0.039
Time to first sips of water (days)	3.66 ± 0.61	4.07 ± 0.77	<0.001
Time to first liquid diet (days)	4.43 ± 1.02	5.03 ± 1.70	0.004
PCT on the 3rd day	0.21 ± 0.18	0.27 ± 0.25	0.083
WBC on the 3rd day(^*^10^∧^9/L)	9.07, 2.52	10.01 ± 2.98	0.025
Highest body temperature within 3 days	37.4, 0.61	37.7 ± 0.61	0.001
**pH value of gastric acidity**			
1d	6.98 ± 0.18	7.02 ± 0.20	0.098
2d	7.10 ± 0.14	7.05 ± 0.20	0.052
3d	7.21 ± 0.21	7.14 ± 0.23	0.060
Gastric drainage	36.65 ± 24.57	52.61 ± 37	<0.001
Hospital stay (days)	9.89 ± 2.46	10.50 ± 2.57	0.114

* *represents a multiplication sign (×)*.

In the postoperative course, there were no significant differences regarding short-term postoperative outcomes between the two groups, including the PCT on the 3rd day after surgery, the pH value of gastric acidity during hospitalization, and postoperative hospital stay, but the time to first flatus (68.60 ± 25.96 h vs. 76.16 ± 21.05 h, *p* = 0.039), time to the first sip of water (3.66 ± 0.61 vs. 4.07 ± 0.77 days, *p* < 0.001), time to first liquid diet (4.43 ± 1.02 vs. 5.03 ± 1.70 days) were significantly shorter in the linear stapler group than that in the circular stapler group.

The indexes, including the highest post-operation body temperature within 3 days (37.4 ± 0.61 vs. 37.7 ± 0.61°C, *p* = 0.001) after the operation and the WBC count on the 3rd day (9.07 ± 2.52 × 10∧9/L vs. 10.01 ± 2.98 × 10∧9/L, *p* = 0.025) and the average gastric tube drainage within 3 days (36.65 ± 24.57 vs. 52.61 ± 37 ml, *p* < 0.001) after surgery, were significantly lower in the linear stapler group when compared with the circular stapler group.

### Postoperative Pathological Results

As shown in Table 3, the number of lymph nodes harvested and the distance of the tumor from resection margins were regarded as important indicators for evaluating the safety of the operation. There were no statistically significant differences in the tumor length (3.52 ± 1.87 vs. 3.42 ± 1.60 cm, *p* = 0.708), the number of lymph nodes harvested (25.77 ± 8.27 vs. 26.99 ± 8.70, *p* = 0.349), positive lymph nodes harvested (3.21 ± 4.65 vs. 2.63 ± 6.97, *p* = 0.508), or TNM stage between the two groups. Rapid pathologic examination during the operation indicated that the surgical margins were negative for cancer cells, and no significant difference was found between the two groups in proximal (5.19 ± 1.63 vs. 5.26 ± 1.35 cm, *p* = 0.779) and distal (3.38 ± 1.79 vs. 3.66 ± 1.77 cm, *p* = 0.317) margins.

**Table 3:** Postoperative pathological results of patients who underwent laparoscopic distal gastrectomy using linear stapler and circular stapler.

**Index**	**Linear anastomosis (*n* = 93)**	**Circular anastomosis (*n* = 80)**	***p*** **-value**
Tumor length (cm)	3.52 ± 1.87	3.42 ± 1.60	0.708
Number of lymph nodes harvested (*n*)	25.77 ± 8.27	26.99 ± 8.70	0.349
Number of positive lymph nodes (*n*)	3.21 ± 4.65	2.63 ± 6.97	0.508
Distance from proximal resection margin to tumor (cm)	5.19 ± 1.63	5.26 ± 1.35	0.779
Distance from distal resection margin to tumor (cm)	3.38 ± 1.79	3.66 ± 1.77	0.317
**Histological grade**			
Poorly differentiated	11	5	0.407
Moderately differentiated	73	65	
Well differentiated	9	10	
**T stage**			
Tis	3	0	
I	28	32	
II	18	9	0.248
III	37	32	
IV	7	7	
**N stage**			
0	47	40	
I	12	16	0.222
II	14	15	
III	20	9	
**TNM Stage**			
I	30	34	0.143
II	34	31	
III	29	15	

### Postoperative Complications

As shown in Table 4, in the linear stapler group, there were 14 cases of postoperative complications, which included 2 cases of the anastomotic complication (1 case of anastomotic bleeding and 1 case of anastomotic leakage), 6 cases of gastrointestinal motility-related complications (3 cases of delayed gastric emptying and 3 cases of inflammatory bowel obstruction), and 6 cases of other complications (3 cases of pneumonia, 1 case of cholecystitis, and 2 cases of lymphatic leakage). Among them, 1 case of severe anastomotic leakage was repaired by a second operation, and 1 case was cured by abdominal cavity washing with saline solution. The three cases of gastroparesis and the three cases of inflammatory bowel obstruction recovered under conservative treatment. Of the 3 cases of pulmonary infection, 1 case was transferred to the intensive care unit (ICU) and fully recovered, 2 cases showed improvement and recovered with anti-inflammatory and symptomatic treatment, 2 cases of lymphatic leakage, and 1 case of cholecystitis improved after conservative treatment.

**Table 4:** Postoperative complications of patients who underwent laparoscopic distal gastrectomy using linear stapler and circular stapler.

**Index**	**Linear anastomosis (*n* = 93)**	**Circular anastomosis (*n* = 80)**	***p*** **-value**
Total	14	16	0.392
Anastomotic complication	2 (14.3%)	4 (25%)	0.657
Bleeding	1	1	
Leakage	1	2	
Stenosis	0	1	
Functional complication	6 (42.9%)	7 (43.75%)	1.000
Delayed gastric emptying	3	5	
Dumping syndrome	0	0	
Inflammatory bowel obstruction	3	2	
Others	6 (42.9%)	5 (31.25%)	0.707
Pneumonia	3	2	
Cholecystitis	1	2	
Lymphatic leakage	2	1	
**Classification of Clavien-Dindo**			
II	12 (85.8%)	14(87.5%)	1.000
III	1 (7.1%)	1 (6.25%)	
IV	1 (7.1%)	1 (6.25%)	

In the circular stapler group, there were 16 cases of postoperative complications, which included 4 cases of the anastomotic complication (1 case of anastomotic bleeding, 2 cases of anastomotic leakage, and 1 case of stenosis), 7 cases of gastrointestinal motility-related complications (5 cases of delayed gastric emptying and 2 cases of inflammatory bowel obstruction), and 5 cases of other complications (2 cases of pneumonia, 2 cases of cholecystitis, and 1 case of lymphatic leakage).

Although the total number of postoperative complications in the circular stapler group was higher than that in the linear anastomosis group, the differences were not statistically significant. According to the Clavien–Dindo classification of postoperative complications, the linear anastomosis group accounted for 85.8% of grade II complications, and grade III and grade IV complications accounted for 7.1%. In the circular anastomosis group, grade II complications accounted for 87.5%, grade III and grade IV complications each accounted for 6.25%, and the difference between the two groups was not statistically significant.

### Postoperative Follow-Up

As shown in Table 5, we performed upper gastrointestinal angiography by the oral administration of 100 ml of ultraviolet at 3 months after the operation in all of the patients. The results showed that the diameter of the stoma in the linear stapler anastomosis group was wider than that in the circular stapler anastomosis group (1.5 ± 0.00 vs. 0.98 ± 0.092 cm, *p* < 0.001), and no anastomotic stenosis occurred. Besides, 6 months after the operation, the average number of meals per day, weight change, quality of life (with reflux, oral medication to control symptoms), and the incidence of dumping syndrome did not differ between the two groups.

**Table 5:** Postoperative follow-up of patients who underwent laparoscopic distal gastrectomy using linear stapler and circular stapler.

**Index**	**Linear anastomosis**	**Circular anastomosis**	***p*** **-value**
	**(*n* = 93)**	**(*n* = 80)**	
Anastomotic width (cm)	1.5 ± 0.00	0.98 ± 0.092	<0.001
**Average number of meals**			
<5 times a day	65 (69. 9%)	49 (61.25%)	0.232
≥5 times a day	28 (30.1%)	31 (38.75%)	
**Weight change**			
<10%	77 (82.8%)	64 (80%)	0.637
≥10%	16 (17.2%)	16 (20%)	
**The symptom of reflux**			
Yes	8 (8.6%)	5 (6.25%)	0.558
No	85 (91.4%)	75 (93.75%)	
**PPI medication**			
Yes	3 (3.2%)	2 (2.5%)	1.000
No	90 (96.8%)	78 (97.5%)	
**Dumping syndrome**			
Yes	8 (8.6%)	2 (2.5%)	0.165
No	85 (91.4%)	78 (97.5%)	

## Discussion

With the development of laparoscopic technology and equipment, laparoscopic surgery has been widely used to treat gastric cancer, which is less invasive and expedites postoperative recovery (17, 20). After laparoscopic radical distal gastrectomy, Billroth II or Billroth II + Braun anastomosis is the most commonly used gastrointestinal reconstruction procedure. Moreover, mechanical anastomosis has become the primary method of gastrointestinal reconstruction because it can significantly shorten the time and effectively ensure the consistency and repeatability of the operation. It has become an indispensable part of laparoscopic surgery. The most used anastomosis methods are circular stapler anastomosis and linear stapler anastomosis (21, 22). However, there is no unified conclusion on the pros and cons of each anastomosis.

Complete tumor resection, thorough lymph node dissection, and reliable gastrointestinal reconstruction are the three essential aspects of radical gastric cancer surgery. Mechanical anastomosis has become an essential part of laparoscopic gastric cancer surgery because it can reduce surgeon workload, shorten the operating time, and reduce the error of human factors. We found that the linear stapler group's operation time and anastomosis time were significantly shorter than those of the circular stapler group. We believed the effect might be caused by the following factors: the use of linear staplers simplified the procedure and reduced operative time compared with circular staplers (23). When using a circular stapler to complete the anastomosis, not only does the stomach wall need to be opened, but the anvil is also placed in the jejunum two times, which makes the process of digestive tract reconstruction relatively cumbersome compared with linear anastomosis. Therefore, the process of completing the reconstruction of the digestive tract with the circular stapler was relatively cumbersome, which prolonged the time of anastomosis and operation to a certain extent.

In the process with circular staplers, a long time of opening the stomach wall would increase the risk of postoperative fever and even abdominal infection due to the exposure of the stomach contents. Therefore, we found through comparative analysis that the white blood cell counts and the highest body temperature within 3 days after the operation of the linear stapler group were lower than those of the round stapler group. Although the PCT in the linear stapler group was lower than that of the circular stapler group on the 3rd day after surgery, the difference was not statistically significant. In addition, the prolonged opening of the stomach wall may increase the risk of tumor cells spreading in the abdominal cavity, so its impact on the long-term survival of patients' needs further research.

After laparoscopic radical distal gastrectomy, the reconstruction of the digestive tract is a crucial evaluation index for the operation's success. Operation's success can promote rapid recovery and ensure an excellent long-term nutritional status and quality of life after surgery. Our study showed that the time of first exhaust and the time to first water intake after the operation of the linear stapler group were earlier than those of the circular stapler group. In the study of Gong et al. (24) the results showed that the first exhaust and the first time of water intake after the operation of the linear stapler group were earlier than that of the circular stapler group, which is consistent with the conclusions of our study. The emptying of the remnant stomach after surgery relies on gravity, so the size of the remnant stomach and gastrointestinal anastomosis is particularly important (25). The patients underwent upper gastrointestinal angiography 3 months after the surgery. The results showed that the diameter of the anastomosis in the linear stapler group was broader than that in the circular stapler group, which promoted the passage of gastric contents. In addition, we found that the gastric tube drainage volume in the linear stapler group was less than that of the circular stapler group at 3 days postoperatively. Therefore, we believed that using the linear stapler could promote the recovery of postoperative gastrointestinal function.

Mechanical anastomosis has become the primary method in laparoscopic radical distal gastrectomy. The literature pointed out that mechanical anastomosis could ensure the safety of surgery and reduce the occurrence of postoperative complications (26). In the Kawamura et al. (21) study, the incidence of anastomosis-related complications in the linear stapler group (0.7 vs. 8.2%, *p* = 0.005) was lower than that in the circular stapler group, especially in the anastomotic leak and postoperative anastomotic stenosis. Although the linear anastomosis group had fewer postoperative complications than the circular one, the difference was not statistically significant, which may be related to insufficient sample size. At the same time, the use of linear staplers still has certain advantages compared with that of circular staplers. (1) The linear stapler was easy and straightforward to operate with a short learning curve. (2) The linear stapler provides three rows of staples, and the circular stapler provides two rows of staples. Therefore, the linear stapler is safer and more reliable (27, 28), reducing anastomosis-related complications (29, 30). (3) The use of a linear stapler had a better visual field, which made it easier for the surgeon to evaluate and control the quality of the operation (30). In addition, studies have shown that the short-term postoperative complications are related to adverse effects on the overall and recurrence-free survival of patients after laparoscopic radical gastric cancer surgery (31–33).

While ensuring the safety of surgery and postoperative recovery, the functional recovery of the digestive tract after reconstruction has become an essential component of short-term postoperative recovery, which has attracted increasing attention from clinicians. Gastroplegia syndrome is considered one of the most common complications related to digestive tract function, with an incidence rate of approximately 2~3% (34). The results of the study showed that the incidence of gastroparesis syndrome in the linear stapler group was much lower than that in the circular stapler group (3.2 vs. 6.2%, *p* = 0.561), but the difference was not statistically significant. Recent studies have revealed that operation time is one factor that affects the occurrence of gastroparesis. In the study, the linear stapler group's operation time and anastomosis time were shorter than those of the circular stapler group. In addition, the circular stapler was inserted in the jejunum through gastrointestinal anastomosis when Braun anastomosis was performed, resulting in gastrointestinal anastomosis edema and gastroparesis.

It is necessary to consider not only the difficulty and economic benefits of the surgery but also the quality of life of the patients in the choice of reconstruction method after distal gastrectomy. Postoperative bile reflux is one of the most critical factors that affect the quality of postoperative life (21, 26). Reflux often causes discomfort, such as upper abdominal pain and heartburn in patients (35). Severe reflux could lead to anastomotic stomatitis and remnant gastritis, which are significant risk factors for remnant gastric cancer (36, 37). Even though the linear stapler group had a wider anastomotic diameter than the circular stapler group, it did not aggravate bile reflux at the anastomosis. It did not affect the emptying of the remnant stomach. There was no statistically significant difference in the patients' quality of life after surgery. However, the follow-up period in this study was relatively short, so the intergroup comparison of the long-term complications, tumor recurrence rate, and survival rates still needed to be further followed up.

Compared with the circular stapler, the linear stapler could be easily placed into the abdominal cavity *via* a trocar, making it possible to complete radical gastric cancer under total laparoscopic surgery. Total laparoscopic distal gastrectomy was first accomplished and reported by Kanaya in 2002, which minimizes the surgical trauma (38) and provides a better surgical field of vision and larger operating space in obese patients. In obese patients, total laparoscopic surgery could reduce the dependence on the length of the incision and, at the same time, avoid problems, such as excessive stretching of the intestine during extra-abdominal anastomosis (39). In addition, studies have shown that total laparoscopic surgery could accelerate the recovery of gastrointestinal function after surgery compared with laparoscopic-assisted and open surgery (15).

There were some limitations in our study: (1) it was a small retrospective study, and the results might be biased. (2) The sample size was relatively small, which might lead to relatively insufficient statistical power. (3) The results were limited by the lack of long-term follow-up owing to the insufficient clinical sample size. Therefore, it is necessary to conduct multicenter, large-scale randomized controlled trials, and heterogeneous cohorts to identify the optimal anastomosis method during total laparoscopic distal gastrectomy.

## Conclusion

In conclusion, Billroth II + Braun anastomosis is vital for gastrointestinal reconstruction procedures after laparoscopic radical distal gastrectomy. Among them, the linear stapler and the circular stapler are safe and feasible for completing the digestive tract reconstruction. However, the linear stapler is compared with circular anastomosis in the terms of shortening the operation time, reducing the dependence on the incision length, and accelerating the short-term recovery after the operation.

## Data Availability Statement

The original contributions presented in the study are included in the article/supplementary material, further inquiries can be directed to the corresponding author.

## Ethics Statement

The studies involving human participants were reviewed and approved by Medical Ethics Committee of Qilu Hospital of Shandong University. The patients/participants provided their written informed consent to participate in this study.

## Author Contributions

DS and RZ wrote the manuscript. WY and MW designed the overall study and completed the surgery. PL, XZ, and YC analyzed the data. YL, YH, and DS completed the follow-up. WY revised the manuscript. WY and YC acquired the funding. All the authors read and approved the final manuscript.

## Funding

The study was supported by the Natural Science Foundation of Shandong Province (Nos. ZR201911030023 and ZR2019LZL006).

## Conflict of Interest

The authors declare that the research was conducted in the absence of any commercial or financial relationships that could be construed as a potential conflict of interest.

## Publisher's Note

All claims expressed in this article are solely those of the authors and do not necessarily represent those of their affiliated organizations, or those of the publisher, the editors and the reviewers. Any product that may be evaluated in this article, or claim that may be made by its manufacturer, is not guaranteed or endorsed by the publisher.
